# High Throughput Interrogation of Somatic Mutations in High Grade Serous Cancer of the Ovary

**DOI:** 10.1371/journal.pone.0024433

**Published:** 2011-09-08

**Authors:** Ursula A. Matulonis, Michelle Hirsch, Emanuele Palescandolo, Eejung Kim, Joyce Liu, Paul van Hummelen, Laura MacConaill, Ronny Drapkin, William C. Hahn

**Affiliations:** 1 Department of Medical Oncology, Dana-Farber Cancer Institute, Boston, Massachusetts, United States of America; 2 Department of Pathology, Women's and Perinatal Pathology Division, Brigham and Women's Hospital, Boston, Massachusetts, United States of America; University of Pennsylvania School of Medicine, United States of America

## Abstract

**Background:**

Epithelial ovarian cancer is the most lethal of all gynecologic malignancies, and high grade serous ovarian cancer (HGSC) is the most common subtype of ovarian cancer. The objective of this study was to determine the frequency and types of point somatic mutations in HGSC using a mutation detection protocol called OncoMap that employs mass spectrometric-based genotyping technology.

**Methodology/Principal Findings:**

The Center for Cancer Genome Discovery (CCGD) Program at the Dana-Farber Cancer Institute (DFCI) has adapted a high-throughput genotyping platform to determine the mutation status of a large panel of known cancer genes. The mutation detection protocol, termed OncoMap has been expanded to detect more than 1000 mutations in 112 oncogenes in formalin-fixed paraffin-embedded (FFPE) tissue samples. We performed OncoMap on a set of 203 FFPE advanced staged HGSC specimens. We isolated genomic DNA from these samples, and after a battery of quality assurance tests, ran each of these samples on the OncoMap v3 platform. 56% (113/203) tumor samples harbored candidate mutations. Sixty-five samples had single mutations (32%) while the remaining samples had ≥2 mutations (24%). 196 candidate mutation calls were made in 50 genes. The most common somatic oncogene mutations were found in *EGFR*, *KRAS, PDGRFα, KIT, and PIK3CA*. Other mutations found in additional genes were found at lower frequencies (<3%).

**Conclusions/Significance:**

Sequenom analysis using OncoMap on DNA extracted from FFPE ovarian cancer samples is feasible and leads to the detection of potentially druggable mutations. Screening HGSC for somatic mutations in oncogenes may lead to additional therapies for this patient population.

## Introduction

Epithelial ovarian cancer is the most lethal of all of the gynecologic malignancies, and new treatments are needed for both newly diagnosed patients as well as patients with recurrent cancer [1]. Within epithelial ovarian cancer, HGSC is the most common subtype and is associated with initial chemotherapy responsiveness when first diagnosed. However, most cancers recur and become increasingly chemotherapy resistant. The success of conventional chemotherapy for the treatment of ovarian cancer has reached a plateau, and new means of molecularly and genetically characterizing ovarian cancer in order to “personalize” and improve treatment are needed [2], [3].

Activating point mutations in proto-oncogenes have been observed in many human cancers, and such mutations can confer ‘oncogene addiction’ upon the relevant cancer cells [4]. This oncogene dependency provides a basis for targeting activated oncogenes in treatment as exemplified by the success of imatinib and erlotinib in cancers that harbor *BCL-ABL* and *EGFR* alterations, respectively. Abundant evidence now indicates that these gain-of-function mutations do not occur randomly within oncogenes, but instead, mutations affecting a relatively small number of codons account for the overwhelming majority of activating events in cancer. For example, single base changes at codons 12, 13 and 61 in *KRAS* mutations comprise the majority of activating oncogenic mutations [5]. Similarly, *BRAF* mutations affecting codon 600 constitute >90% of melanoma *BRAF* mutations; genetic changes in an additional 10–12 codons account for most of the remaining cancer-associated *BRAF* mutations identified to date [6], [7].

To identify these oncogenic mutations in archival tissues, we have adapted a high-throughput genotyping platform to determine the mutation status of a large panel of known cancer oncogenes [8], [9]. Specifically, we have developed a mutation detection protocol, termed OncoMap, which employs mass spectrometric-based genotyping technology (Sequenom) to identify oncogenic mutations. The current version of this protocol is able to detect more than 1000 mutations in 112 commonly mutated genes in both fresh frozen and paraffin-embedded tissue samples. This report describes our successful application of OncoMap to a cohort of patients with advanced HGSC in order to identify oncogenic mutations.

## Results

In the initial OncoMap analysis, 56% (113/203) tumor samples harbored candidate oncogenic mutations. Sixty-five samples had single mutations (32%) while the remainder had ≥2 mutations (24%). In total, 196 candidate mutation calls were made in 50 genes.

The most commonly mutated oncogenes were *EGFR* (9.4%), *KRAS* (4.5%), *PDGFRα* (4.5%), *KIT* (3.0%), and *PIK3CA* (3%); others that were less commonly mutated included: *BRAF* (1%), *CUBN* (0.5%), *and NRAS* (2.5%). We also identified mutations in many other genes at lower frequencies including: *ABL1* (2.5%), *STK11* (2.5%), *EPHA1* (2%), *RET* (1.5%), *SMARCB1* (1.5%), *ATM* (1%), *FLT3* (1%), *MLL3* (1%), *MYC* (1%), *NF2* (1%), *NOTCH1* (1%), *NTRK1* (1%), *PIK3R1* (1%), *ROBO2* (1%), *APC* (0.5%), *FES* (0.5%), *FYN* (0.5%), *GATA1* (0.5%), *NF1* (0.5%), *NTRK3* (0.5%), *PALB2* (0.5%), *PKHD1* (0.5%), *PTEN* (0.5%), *RUNX1* (0.5%), *SMO* (0.5%), *SPTAN1* (0.5%), and *TSHR* (0.5%).

The most common somatic mutation identified involved the tumor suppressor gene *TP53*, which was detected in 24.8% of the samples. Since OncoMap interrogates only a subset of *TP53* mutations and does not detect deletion events, the observed frequency of *TP53* alterations agrees with recent work from The Cancer Genome Atlas Project (TCGA) [10] that has confirmed the finding that *TP53* mutations are the most common somatic mutation in HGSC cancers. In addition, we identified mutations in other tumor suppressor genes including *RB1* (3%) and *VHL* (3.5%).

Somatic mutations were then validated by hME, and the following ones were validated: *EGFR*, *HRAS*, *KRAS*, *NRAS*, *PIK3CA*, *BRAF*, *RB1*, *TP53*, *ATM*, *CUBN*, and *FLNB*. Table S1 lists the validated mutations found in our cohort of HGSC.

## Discussion

Our group has demonstrated that somatic oncogene mutations can be detected in HGSC using a Sequenom based assay called OncoMap that uses DNA derived from FFPE tissue. Although HGSC is characterized by gene copy number changes [11], low frequency mutations in a number of oncogenic genes were found in 56% of the cancers in our 203 sample cohort, and many of these mutations are potentially druggable using novel biologic agents. Most mutations were found in low frequency, and most specific mutations were found in fewer than 5% of samples. Validation using hME was performed on genes of interest, and several important genes were found to be mutated; all mutations were not validated because of cost and level of interest. In clinical practice, we anticipate that all mutations identified by OncoMap profiling will be validated in CLIA-approved laboratories.

Thus, OncoMap which uses Sequenom technology is able to inexpensively screen for multiple mutations using DNA extracted from FFPE samples in cancers such as HGSC that have multiple mutations present in low frequency. Other advantages of OncoMap include the ability to rapidly expand the “hotspot” mutation library as additional mutations are discovered and new novel biologic agents are successfully tested. Limitations of OncoMap include that only “hotspot” mutations are located and that validation of mutations is necessary; other mutations not included in the OncoMap panel will be missed. Although whole exome or whole genome sequencing is now possible in research laboratories, the routine use of these technologies in paraffin embedded samples is not possible. Thus, OncoMap provides a rapid, reasonable cost method to identify oncogenic mutations in human cancer specimens.

The clinical implications of somatic mutations in HGSC are unknown and will need to be further investigated. Somatic mutations in cancers can lead to constitutive activation of signaling pathways that are normally activated by activated growth factor receptors, and these mutations can lead to overall genomic instability [12]. Alterations in gene copy number and gene expression have both been demonstrated to be important in ovarian cancer, while mutations have been felt to be less important [11].

Several mutated oncogenes of interest were found in our cohort of HGSC samples tested and analyzed with OncoMap. *EGFR* was found to harbor mutations in close to 10% of cases, and EGFR inhibitors such as erlotinib could be tested in this subset of cancers. In lung cancer, these inhibitors are used to treat cancers that harbor exon 20 variants, codon 719 variants, and L858R substitutions in addition to other types of *EGFR* mutations [13], [14]. We identified HGSC with a codon 719 variant which were validated by hME. Thus, testing of EGFR inhibitors appears warranted when EGFR mutations are detected. EGFR inhibitors have been tested in ovarian cancer with response rates of 10% or less [15]–[17]; however, none of these studies prospectively tested ovarian cancers for EGFR mutations, a practice now routinely done for non-small cell lung cancer that has resulted in the molecularly targeted use of EGFR inhibitors. *EGFR* mutations and expression was tested for retrospectively in Schilder et al, and a partial response was observed in 1 patient who did have an EGFR mutation [17].

Our rate of *PIK3CA* mutations of 3% found in HGSC parallels that found by the Sanger Center [18]. Other groups have reported low rates of both *AKT* and *PIK3CA* mutations but higher frequency of gene amplification for *PIK3CA* [19]. Inhibitors of the PI3kinase pathway are currently being studied in ovarian cancer, and activity of these agents has been reported in ovarian cancer [20], [21]. For example, MK2206, an AKT inhibitor, was tested in a Phase 1 study in patients with advanced solid tumors. All 3 ovarian cancer patients who were enrolled in this study demonstrated a decrease in their CA125 levels, suggesting anti-tumor activity of MK-2206 in ovarian cancer. GDC0941, a PI3kinase inhibitor, has also demonstrated activity in ovarian cancer specifically in situations of *PIK3CA* amplification. With the development of additional inhibitors of the PI3kinase pathway and because of anti-cancer activity of these agents in ovarian cancer, identification of aberrations of this pathway will become increasingly important in HGSC.

Other validated genes of interest found in our study include *BRAF*, *KRAS*, *HRAS*, and *NRAS*, and all of these genes have available biologic agents that could target the effects of these oncogenic mutations. *TP53* mutations are found commonly in ovarian cancer [22], and our data supports and parallels this data.

This work corroborates the recently published TCGA data [10]; future studies will be necessary to correlate the presence of these mutations with biologic activity and prognosis of the cancer and whether these mutations predict anti-cancer activity of targeted biologic agents. In addition, correlating somatic mutations with other objective assessments of the genetic make up of cancers such as gene expression profiling and gene copy number will be vital to understanding a more complete genetic picture of HGSC.

## Materials and Methods

### Patients and patients' samples

Pathology records were reviewed between 1999 and 2004 from the Division of Gynecologic Pathology at the Brigham and Women's Hospital in Boston MA, and International Federation of Gynecology and Obstetrics (FIGO) stage III or IV HGSC ovarian cancer cases were selected. The Dana-Farber/Harvard Cancer Center Institutional Review Board (IRB) granted approval to collect FFPE samples. Because all of the samples were de-identified, the IRB granted us a waiver to collect the samples without patient consent.

FFPE samples were reviewed by a gynecologic oncology pathologist (MH) who reviewed pathology reports as well as FFPE tissue blocks and selected the areas of highest percentage of cancer that were eventually cored for DNA extraction. Patients with known BRCA germline mutations were excluded in this set and are being studied in another data set. A total of 203 samples were selected.

### DNA extraction and quantification

Genomic DNA was extracted from the cored FFPE patient tissue samples with QIAamp DNA FFPE Tissue Kit (Qiagen) according to the manufacturer's protocol. Briefly, cores were deparaffinized in xylene and further lysed in denaturing lysis buffer containing proteinase K. The tissue lysate was incubated at 90°C to reverse formalin crosslinking. Using QiaCube, the lysate was applied to the DNA binding column and the column was washed serially, and then eluted in 30 ul of distilled water. Genomic DNA was quantified using Quant-iT PicoGreen dsDNA Assay Kit (Invitrogen) per manufacturer's protocol. 250 ng of genomic DNA was used for the analysis.

OncoMap v3.0 was performed on all samples, and the genes and number of mutations tested for in this version of OncoMap version are listed in Table 1. Initially, primers were designed that enable mutation detection. Tumor-derived genomic DNA was subjected to whole genome amplification. Next, multiplexed PCR was performed on tumor genomic DNA to amplify regions harboring loci of interest, or ‘query’ nucleotides. After denaturation, PCR products were incubated with oligonucleotides that anneal immediately adjacent to the query nucleotide, and a primer extension reaction was performed in the presence of chain-terminating di-deoxynucleotides that generate allele-specific DNA products. Primer extension products were spotted onto a specially designed chip and analyzed by MALDI-TOF mass spectrometry to determine the mutation status. Since allele (or mutation) calling depends exclusively on the mass of the resulting primer extension product, the Sequenom assay does not require expensive fluorescence primer labeling and has a very low error rate. The cost of solely running the OncoMap mutational assay is approximately $200 per sample independent of the number of samples run.

**Table 1:** Known oncogenic mutations tested for in OncoMap.

Gene Name	Number of Mutations	Gene Name	Number of Mutations	Gene Name	Number of Mutations
ABL1	7	FES	3	PALB2	3
ALB2	1	FGFR2	1	PDGFRA	11
ADAMTSL3	4	FGFR3	6	PDGFRB	3
ALK	7	FGFR4	3	PDPK1	2
AML1/RUNX1	8	FLNB	5	PIK3CA	25
APC	1	FLT3	12	PKHD1	5
AR	2	FMS	3	PTCH	6
ATM	3	FYN	3	PTEN	10
ATP8B1	3	GATA1	16	PTPN11	14
AURKA	3	GNAS	3	RAF1	2
AURKB	1	GUCY1A2	3	RB1	2
AURKC	3	HRAS	1	RET	2
AXL	1	IGF1R	5	RET	12
BMX	1	JAK2	3	ROBO1	2
BRAF	7	JAK3	1	ROBO2	4
BRCA1	3	KIT	13	ROS1	4
BRCA2	6	KRAS	5	SIX4	4
BUB1	2	LRP1B	12	SMAD2	3
C14orf 155	3	LYN	1	SMAD4	4
CDH1	5	MADH4	7	SMARCB1	9
CDKN2A	6	MAP2K4	13	SMO	3
CEBPA	13	MEN1	6	SPTAN1	4
CREBBP	2	MET	5	STK11	7
CTNNB1	16	MLL3	5	SUFU	3
CUBN	3	MPL	3	TBX22	3
DBN1	2	MSH2	2	TCF1	2
DDR1	2	MSH6	3	TEC	1
DDR2	1	MYC	15	TFDP1	2
EGFR	62	MYH1	3	TIAM1	4
EPHA1	2	NF1	5	TIF1	3
EPHA3	18	NF2	11	TP53	11
EPHA4	4	NOTCH1	10	TRIM33	4
EPHA5	6	NPM1	6	TSC1	2
EPHA8	1	NRAS	7	TSHR	5
EPHB1	10	NTRK1	1	VHL	8
EPHB6	5	NTRK1	1	WT1	2
ERBB2	2	NTRK2	1		
FBXW7	8	NTRK3	6		

Once mutations were identified, validation was performed on a selected subset of mutations using the multi-base hME extension chemistry as described previously [8], [9]. Primers and probes were designed using Sequenom MassARRAY Assay Design 3.0 software, applying default multi-base extension parameters but with the following modifications: maximum multiplex level input adjusted to 6; maximum pass iteration base adjusted to 200.

## Supporting Information

Table S1 **Validated Mutations by hME.** This table lists the validated mutations found in our cohort of HGSC. Validation was performed by hME.
(DOC)
