# Supplementary material for: High Throughput Interrogation of Somatic Mutations in High Grade Serous Cancer of the Ovary
Source: PLoS One. 2011 Sep 8;6(9):e24433. doi: 10.1371/journal.pone.0024433 (PMC3169600; doi:10.1371/journal.pone.0024433)
Supplement: Table S1 — Validated Mutations by hME. This table lists the validated mutations found in our cohort of HGSC. Validation was performed by hME. (DOC) [file pone.0024433.s001.doc]

| Sample | Mutation(s) validated |
| --- | --- |
| G99-58861-13 | TP53_R273H |
| G99-57771-5 | TP53_G245S |
| G99-51613-13 | TP53_R175H |
| G04-64805-9 | TP53_R273H |
| G04-62462-9 | TP53_Y220C |
| G04-57502-9 | TP53_R248Q |
| G04-55153-7 | TP53_R175H |
| G04-50723-9 | PIK3CA_C420R |
| G03-59771-11 | TP53_R273H |
| G03-59581-13 | TP53_R175H; FLNB_R566Q |
| G03-55270-15 | TP53_R248W |
| G03-54065-8 | EGFR_G719C |
| G02-64331-18 | TP53_R306*, CUBN_I3189V |
| G02-61944-19 | TP53_Y220C |
| G02-52303-6 | TP53_Y220C |
| BG-05-T71228-C2 | KRAS_G12V |
| BG-05-A71856-A4 | TP53_Y220C |
| BG-05-A63837-D5 | TP53_G245S |
| G99-58861-22 | TP53_R273H |
| G99-58730-2 | KRAS_G12A |
| G99-51613-4 | TP53_R175H |
| G99-50999-10 | TP53_R175H |
| G95-39055-2 | TP53_R273H |
| G04-64805-2 | TP53_R273H |
| G04-62462-2 | TP53_Y220C |
| G04-55153-2 | TP53_R175H |
| G04-50723-36 | PIK3CA_C420R |
| G03-59771-3 | TP53_R273H |
| G03-59581-17 | TP53_R175H; FLNB_R566Q |
| G03-55270-3 | TP53_R248W |
| G02-64331-22 | TP53_R306*; CUBN_I3189V |
| G02-61944-2 | TP53_Y220C |
| G02-50254-7 | EGFR_G719C |
| G01-52761-8 | HRAS_G12V |
| BG-05-A71856-D1 | TP53_Y220C |
| BG-05-A63837-C1 | TP53_G245S |
| G99-61653-11 | KRAS_G12V |
| G99-57906-15 | TP53_R273H |
| G99-56917-16 | TP53_R248Q |
| G04-58410W-10 | TP53_R273H |
| G04-55430B-7 | TP53_R175H |
| G03-57126-4 | TP53_R248W |
| G03-51896-4 | BRAF_N581S |
| G02-52786-3 | RB1_R320*; TP53_R175H |
| G02-50916-6 | KRAS_G12V |
| G01-64614-25 | KRAS_G12D |
| G01-51270-5 | TP53_R273C |
| G00-58242-4 | TP53_G245S |
| G00-58063-13 | TP53_R175H |
| BG-05-X60765-A6 | TP53_R213* |
| BG-05-R63072-B1 | ATM_P604S |
| BG-05-G64570-F5 | TP53_R175H |
| G00-58242-2 | TP53_G245S |
| G00-58063-7 | TP53_R175H |
| BG-05-G64570-B1 | TP53_R175H |
| BG-05-D74977-C1 | NRAS_Q61R |
| G04-55153-5 | TP53_R175H |
| G04-64805-7 | TP53_R273H |
| G06-66497-C9 | TP53_Y220C |
| G07-74175-D5 | ATM_P604S |
| G08-81645-A13 | TP53_R248Q |
| G02-55757 -5 | KRAS_G12V |
